# Hemorrhagic Cerebrovascular Pathology in the Pediatric Population

**DOI:** 10.3389/fneur.2020.01055

**Published:** 2020-09-17

**Authors:** Waldo R. Guerrero, Sudeepta Dandapat, Santiago Ortega-Gutierrez

**Affiliations:** ^1^Department of Neurosurgery, University of South Florida Morsani College of Medicine, Tampa, FL, United States; ^2^Department of Neurology, Radiology, and Neurosurgery, University of Iowa Carver College of Medicine, Comprehensive Stroke Center, Iowa City, IA, United States

**Keywords:** cavernous malformation, developmental venous anomaly (DVA), aneurysm, arteriovenous malformations, pediatric

## Abstract

Hemorrhagic cerebrovascular disease in the pediatric population can have devastating and long-term effects. Progress in the fields of genetics, neuroimaging, pharmacology, and surgical techniques has led to improved diagnosis and management of pediatric cerebrovascular diseases. In this review we discuss the current etiologies and medical and surgical treatments of hemorrhagic cerebrovascular pathology affecting infants and children. A special emphasis is placed on neuroendovascular treatment options. Increased knowledge about this unique pathology and the medical and therapeutic options will empower practitioners to more quickly and accurately identify and accurately treat hemorrhagic diseases in the pediatric population.

## Pediatric Hemorrhagic Cerebrovascular Disease in Infants and Children

The incidences of non-traumatic subarachnoid hemorrhage (SAH) and intracerebral hemorrhage (ICH) in pediatric patients are 0.4/100,000/year and 0.8/100,000/year (1). Most cases of SAH and ICH in the pediatric population are a result of a secondary pathology such as a hematological condition, tumor, ruptured vascular malformation, or cerebral infection [Table 1; (2, 3)]. In this section we will focus on the most common non-traumatic vascular causes. Of note vascular malformations might account for 17.5–73.5% of pediatric ICH [Table 1; (4–6)].

**Table 1:** Most common etiologies of spontaneous intracerebral hemorrhage in children.

Vascular malformations (50%)
Arteriovenous malformations (39%)
Cavernous malformations (11%)
Bleeding diathesis (21%)
Coagulopathies: liver failure, DIC, congenital
Thrombocytopenias: malignant (ALL, AML), congenital (aplastic anemia, bone marrow failure), immune mediated, autoimmune
Aneurysm (9%)
Hemorrhagic primary intracranial tumor (6%)
Other (10%)
Hemorrhagic CNS infection
Cerebral vasculitis
Moyamoya disease
Illicit drug abuse


## Section 1: Arteriovenous Malformations (AVMs)

The presence of arteriovenous (AV) shunting through a nidus of coiled tortuous vascular connections that lack of capillaries and connect feeding arteries to draining veins are the hallmark of arteriovenous malformations (AVMs) (7). AVM incidence is estimated to be 1 per 100,000 per year (8). Although AVM incidence is lower in kids, they tend to rupture more frequently than in the adult population (9–13) and are the most frequent cause of ICH in the pediatric population (14). Although the exact pathophysiology remains to be elucidated, it is thought that the majority of AVMs develop during the 3rd week of embryogenesis secondary to persistence of development of a new AV connection form a presumed spontaneous mutation. AVMs have also been associated with specific genetic syndromes Table 2. However, mutations in RASA-1 have been associated with AVMs in a small number of families (15).

**Table 2:** AVM-associated syndrome genetics.

**Syndrome**	**Mutated gene**	**Gene product**	**Molecular mechanism for AVM formation**	**Mode of inheritance**
Hereditary hemorrhagic telangiectasia (HHT)				
HHT1	ENG			Autosomal dominant
HHT2	ACVRL/ALK1			
Juvenile polyposis-HHT overlap	SMAD4			
Capillary malformation-arteriovenous malformation	RASA1	RAS p21 protein activator	Abnormal angiogenic remodeling of primary capillary plexus	Autosomal dominant/sporadic in 30%
Parkes-Weber's syndrome	RASA1	RAS p21 protein activator	Abnormal angiogenic remodeling of primary capillary plexus	Autosomal dominant/sporadic
Wyburn-Mason's syndrome	NA	NA	Unknown	Sporadic

AVMs are usually diagnosed after ruptured with identification of a hematoma on a CT. MRI and MR/CT angiography are the imaging modalities used to characterize the precise location and the therapeutic planning. DSA remains the gold standard to define the AVM architecture and assessing high risk features such as aneurysm and or venous stenosis or ectasias (16). It is important to recognize that DSA catheter angiogram might miss some components or even the entire AVM when performed in the acute setting of hemorrhage due to the compression of the AVM nidus by the hematoma (17).

**Figure 1:**
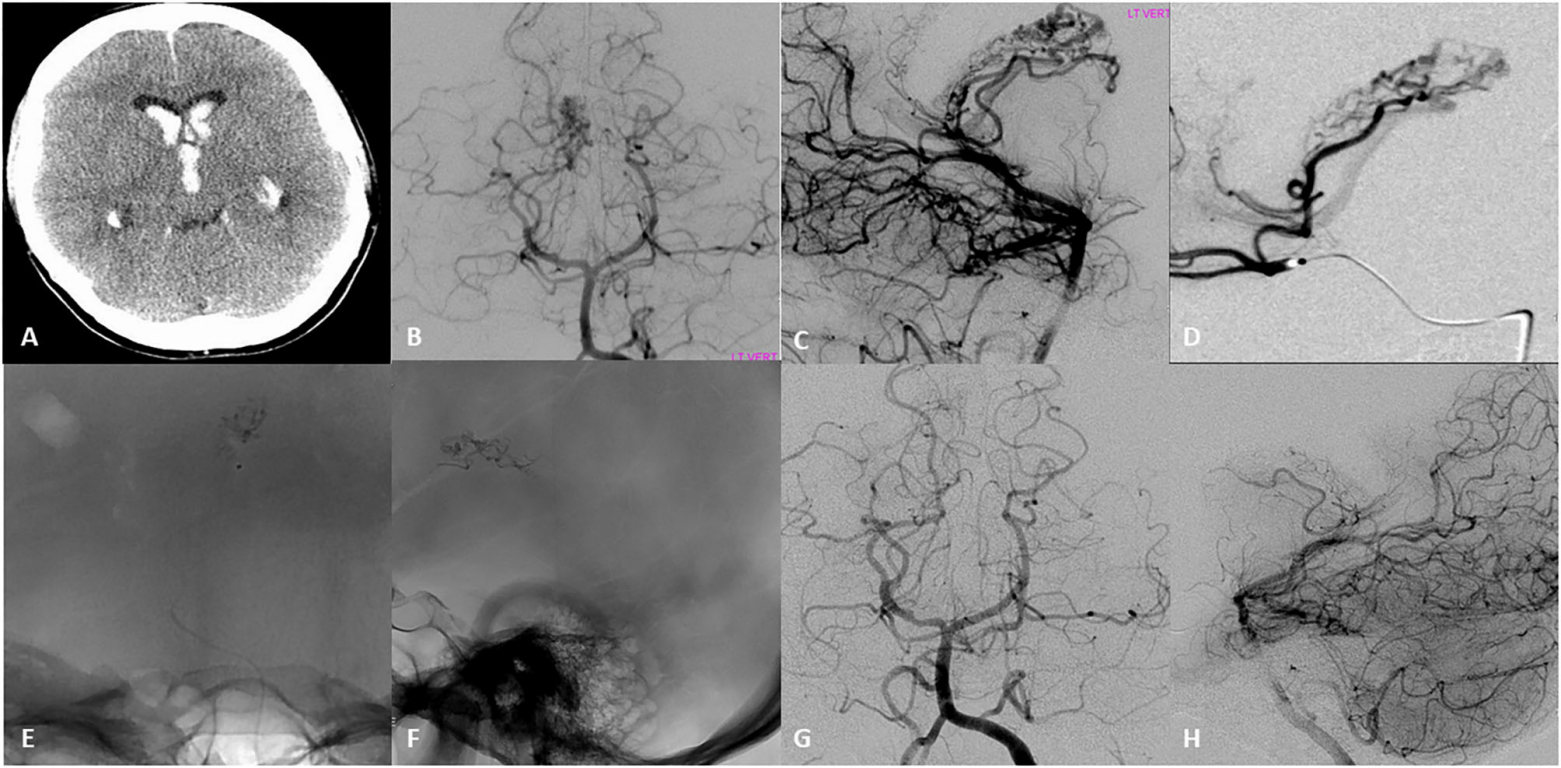
Seventeen-year-old right-handed healthy girl that presented with initial headache nausea and vomiting, that progress to decrease level of consciousness within hours. **(A)** Initial head CT revealed intraventricular hemorrhage in the lateral ventricles and third ventricle, for which she underwent emergent ventriculostomy. **(B,C)** Initial DSA AP and lateral left vertebral artery injection revealed an arteriovenous malformation with a compact nidus located in the lateral ventricle. Arterial feeders arise from the post-erolateral and post-eromedial choroidal arteries, both branches of the right posterior cerebral artery. The drainage occurred into the right internal cerebral vein. **(D)** Superselective DSA examination showed a medial posterior choroidal artery that appeared to feed the nidus of the AVM with opacification of the right internal cerebral vein. **(E,F)** AP and Lateral native images demonstrate the N-Butyl cyanoacrylate (NBCA) cast into the nidus after microcatheter embolization of two pedicles. **(G,H)** DSA Ap and lateral 6–month follow up showed with complete obliteration of the AVM.

AVMs have an annual hemorrhage rate that is thought to be between 2 and 4% with a mortality up to 25% per each event (14). This risk is elevated during the first 5 years post-diagnosis (18–20). The risk of rupture or re-rupture is also higher in the presence of high-risk features such as infratentorial or deep-seated AVMs, deep venous drainage, female sex, associated aneurysms and diffuse AVM morphology (21–26). Given the longer presumed life expectancy and higher risk, conservative management is largely abandoned, despite the natural history not being fully understood in this population. The risk or rupture or re-bleeding persist until the AVM is completely obliterated (27–29), thus the ultimate goal of treatment is to obtain complete angiographic abolition of the AVM with none or minimal neurological sequelae. Treatment options include embolization, stereotactic radiosurgery, and surgical resection. Often a combination treatment is needed. Because of this, the decision to treat pediatric AVMs must include a multidisciplinary team with a neurointerventionalist with expertise in pediatric, a vascular surgeon with expertise in pediatrics, and a radiation oncologist. Although data in this population is still lacking, surgical resection remains the gold standard when feasible and safe (30). It has the potential of offering immediate cure as a single modality or in combination, while also providing evacuation of the hematoma in the acute settings. Various cases series reports obliteration rates between 67 and 100% for children presenting with Spetzler-Martin grades 1–3, with a mortality and morbidity of ~5% respectively. Embolization results are modest when used as a single modality with obliteration rates up to 21% and complication rates up to 7% when performed in high volume centers [Figure 1; (31, 32)]. However, embolization is usually utilized as an adjunct treatment option to resection to decrease the risk peri-operative hemorrhage. Resection is usually done within 24–48 h from embolization thus reducing the risk of intraparenchymal hemorrhage that can occur from rapid rerouting of blood flow from a (now resected) low-resistance AVM into higher resistance normal vasculature (unused to handling the high flow previously running through the AVM), the so-called “normal perfusion breakthrough syndrome” (33). Only a few studies have demonstrated the efficacy and safety profile of radiosurgery as a single modality, achieving obliteration rates between 43 and 80% with neurological complications around 4% (29, 34). Higher-grade lesions carry a lower chance of successful treatment with all modalities but in reference to radiosurgery the rate of successful treatment is (≈35%) (35). However, before its standard application, long term follow-up is required to understand the effects of ionizing radiation on the developing nervous system and the incidence of intracranial malignancies and neuropsychological retardation in children (36, 37). In all cases of treatment, long-term evaluation is critical because recurrence rates can be as elevated as 11% (38, 39). Usually a perioperative and 1-year post-operative DSA are done. This is surveillanced by MRI/MRA annually for the subsequent 5 years (33, 39, 40).

## Section 2: Arteriovenous Fistulas (AVFs)

Arteriovenous fistulas (AVFs) are direct arterial to venous connections deprived of intervening capillaries. Unlike AVMs, AVFs do not contain nidus. Two groups of AVFs exist based on the location of the arterial-venous connection: pial and dural. AVFs can be acquired or congenital. The specific genetic patterns of these lesions are still not well-understood; however, numerous molecules have been implicated in the pathogenesis including vascular endothelial growth factor, sonic hedgehog, upstream-transcription factor II, notch chicken ovalbumin, and the ephrin family. Congenital pial AVFs have been linked to various congenital diseases, the commonest of which is hemorrhagic hereditary telangiectasia which co-presents with AVFs in one-quarter of patients (41). Recent data suggest that ≈9% of patients with AVFs will harbor known mutations (41–43). RASA-1– and HHT-related mutations (ENG and ACVRL1) were most commonly associated with clinical phenotypes including cranial lesions, hypercoagulable state, spinal AVF/AVM, capillary hemangioma (in RASA-1) (15, 41–43).

AVFs can also be acquired. Sinus hypertension and sinus thrombosis have been implicated in the development of dual AVFs (44). Dural AVFs have been seen in patients with hypercoagulable states leading to sinus thrombosis (45). Furthermore, trauma has been shown to be associated with AVFs.

The estimated prevalence of pial AVFs ranges between 0.1/100,0000 and 1/100,000 (46–49) accounting for ~4% of cerebral vascular malformations in the pediatric population (31, 49) and normally presenting between that ages of 3–15 years of age (31). Dural AVFs are more common in adults and its prevalence is not well-studied in the pediatric population. AVFs have a high risk of hemorrhage secondary to high flow (50), with a reported risk of hemorrhage of 1.5–10% annually depending on particular factors such as fistulas at the vein of Galen, petrosal, or straight sinus, extensive cortical venous drainage, and venous varices (51). Weon et al. evaluated 41 patients in the pediatric age group with pial fistula and found the mean age of presentation was 24 months. Out of 41 pediatric patients 7 presented with cerebral hemorrhage (17.1%) (48).

Prenatal diagnosis of AVFs usually occurs using fetal ultrasonography (52, 53). AVFs in the pediatric patient can manifest with high output cardiac failure, non-obstructive hydrocephalus, developmental delay, macrocrania, cognitive impairment, seizures, or focal neurological deficits resulting from large venous varices with mass effect (48, 52–54). Infants with congestive cardiac failure exhibit high-flow fistula demonstrate tachycardia, respiratory distress, poor systemic perfusion, and cyanosis. An audible murmur or cranial might be auscultated (55). Venous congestion may lead to non-obstructive hydrocephalus secondary to venous hypertension.

MRI has limitations in the diagnosis of AVFs but can be used to evaluate for cerebral or spinal cord edema, dilated veins related to venous reflux, or ischemic infarct or atrophy. Catheter angiography is the gold standard diagnostic test where the presence of an AV shunting is demonstrated (56–58). Suspicion of an AVF warrants a 5-vessel angiogram to include the examination of the external carotid artery or all spinal radicular vessels. Venous outflow and the presence of stenosis of the venous outflow, arterial feeders, and the severity of arterial “steal” are all evaluated during cerebral angiography.

Treatment should be targeted at high flow symptomatic lesions (47, 48, 52, 53, 59). Smaller slower flow dural AVFs lesions with minimal or no symptoms may be followed long term as these may spontaneously close (51, 60, 61). However, there is some controversy in the treatment of already profoundly impaired infants (48, 62–64) may not be warranted for infants with microcephaly, extensive ischemic injury, or multiorgan failure.

**Figure 2:**
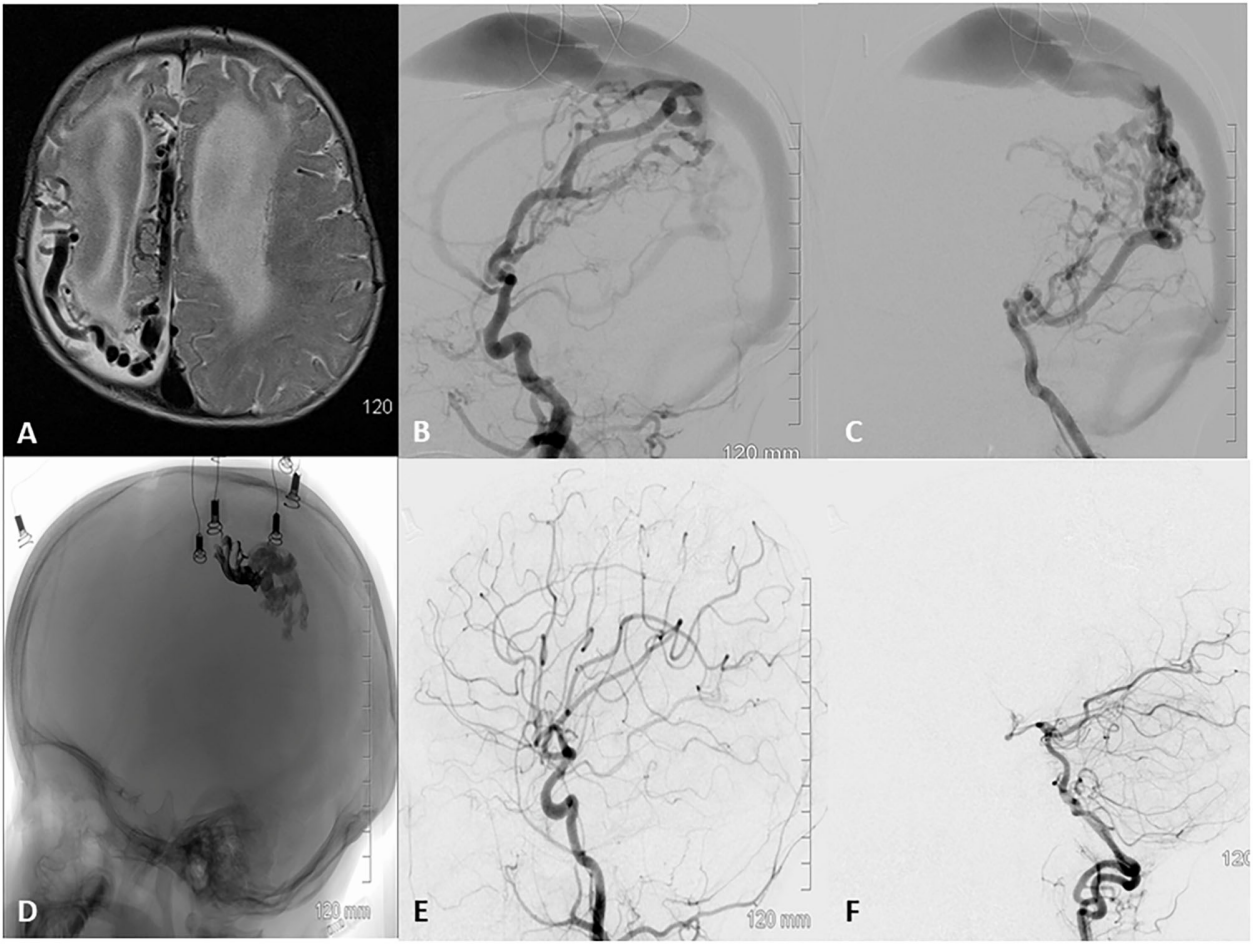
A 36 week old baby was diagnosed with a vascular malformation at 16 weeks of age. The mother noticed abnormal shape of the head with a bulge on the back of right side at 9 weeks and decreased movement of the left hand compared to the right at 16 weeks of age. The right side of the cranial vault is poorly developed, anterior fontanelle is open, full, and pulsating well. **(A)** MRI revealed numerous enlarged flow void in the midline and right hemisphere, with hydrocephalus and hemiatrophy on the right side of the brain. **(B,C)** DSA lateral view runs from the right common carotid and left vertebral revealed a pial AVF with arterial feeders arising primarily from the anterior and posterior branches of the right middle cerebral artery and the parieto-occipital branch of the posterior cerebral artery. The fistulas were multi holed and all were drained by a single enlarged venous channel that drained into the superior sagittal sinus. **(D)** Native lateral skull view demonstrating the final Onyx (darker anterior portion) and NBCA cast sealing several fistulae points over three staging embolization. **(E,F)** DSA 6 month follow up after last embolization show complete obliteration of the pial AVF with restoration of normal arterial phase.

Endovascular techniques have become the first line method for treatment of pediatric AVFs (31, 51, 59, 65). Pial AVFs are more frequently treated transarterially whereas dural AVFs via transvenous. The introduction of the Onyx (ethylene vinyl alcohol copolymer) liquid embolic agent in conjunction with detachable coils has increased the therapeutic efficacy of endovascular approaches for AVFs (41, 42, 51, 59, 66). The objective of the treatment is to obliterate the connection between the arterial feeders and the vein, known as a fistula point. AVF treatment has a high rate of lesion obliteration (86%) and children 2 years of age or older. Good clinical outcome is observed in 72%. However, children <2 years of age have increased complication rates and often require more than one more procedure due to the limitations of contrast and radiation (67, 68). Venous infarction, migration of embolic agents, guide wire rupture of vessels, post-treatment thrombosis of veins, hyperperfusion syndrome, and hydrocephalus are all complications of AVF treatment (41, 42, 51, 69) with a risk of major complications and death between 5 and 10% [Figure 2; (46, 68, 70)].

Microsurgical resection is usually reserved for cases when the risk of embolization of eloquent brain cortex is high (41, 42, 71). Pial AVFs have a higher chance of cure when combined endovascular and open surgical approaches (71%) are used. However, dural AVFs have a high likelihood of cure with endovascular treatment alone (85%) (41, 42).

## Section 3: Vein of GALEN Malformations

**Figure 3:**
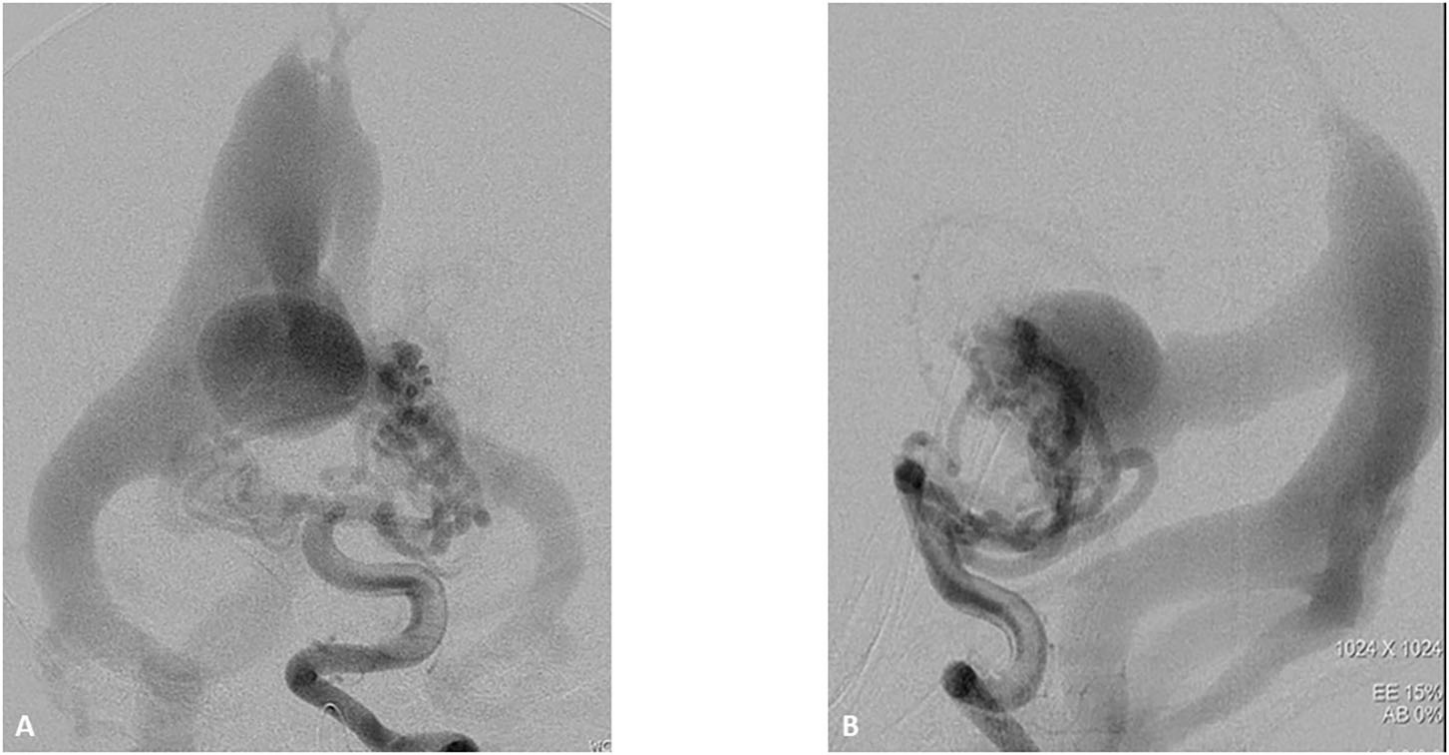
One-month boy presented with severe high failure requiring pressors and ICU management. Initial ultrasound through the frontal fontanelle showed concerns for a vein of Galen malformation. A vertebral DSA AP **(A)** and lateral **(B)** views reveal a high flow vein of Galen choroidal malformation with enlarged choroidal feeders of the posterior cerebral artery, left greater than right. These fistulae drain into a dilated vein of Galen and median vein of the prosencephalon. There is filling of the bilateral transverse sigmoid sinuses, right greater than left as well as some opacification of the occipital.

**Figure 4:**
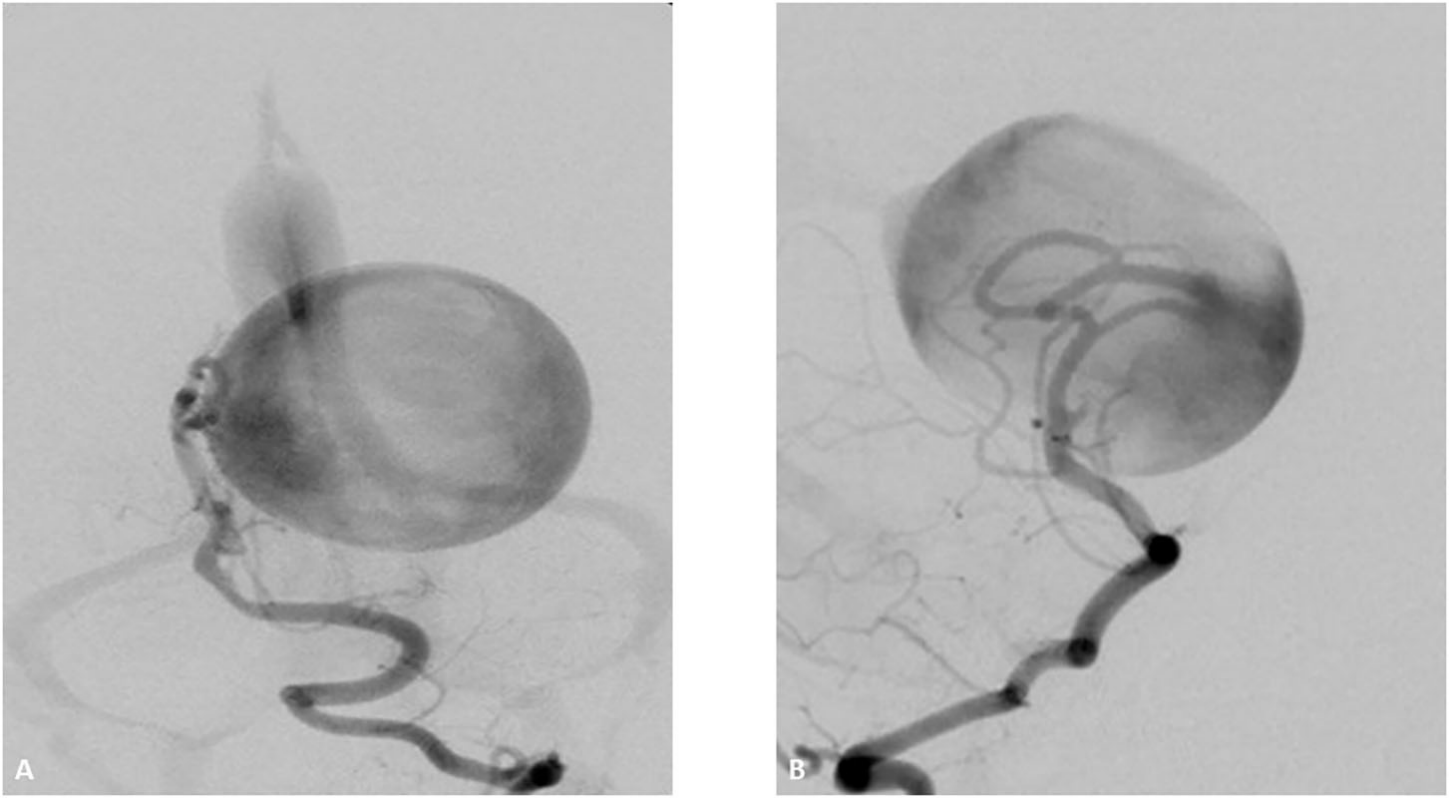
Two-month-old boy presenting with respiratory distress and increase obtundation. Vertebral DSA AP **(A)** and lateral **(B)** show a high-flow Mural type Vein of Galen Malformation with feeders arising from the right posterior cerebral artery and draining in to the anterolateral wall of a dilated vein of Galen. There is mild stenosis of the median prosencephalic vein decreasing the venous shunt. The transverse and sigmoid sinuses have normal appearance.

VOGM is a subtype of dural fistula that results from an abnormal regression of midline venous structures the medial prosencephalic vein of Markowski, which is the precursor of the vein of Galen (72, 73). Arteriovenous shunts from arteries that connect with the anterior segment of the median prosencephalic vein, which further enlarges under the stress of high flow from the choroidal arteries. Two types of VOGM, choroidal, and mural (73), exist. The choroidal type consists of various feeders including thalamoperforating, pericallosal arteries, and anterior and posterior choroidals in the subarachnoid space in the choroidal fissure and is usually found in neonates with severe symptoms (Figure 3). The mural type fistulas can be single. Although more commonly they are multiple and congregate into a single venous chamber (Figure 4). They usually present with macrocephaly secondary to hydrocephalus. Mixed types can be also seen (74).

Ultrasonography is used to assess VOGM flow in an ill neonate although a large procencephalic vein can be seen during the third trimesters of gestation by ultrasound. MRI of the brain can evaluate the brain for cerebral atrophy or ischemia that may be secondary to a CBF steal phenomenon. Cardiac function is assessed using echocardiography. Echocardiogram may show a dilated superior vena cava, dilated innominate vein, and right ventricular dilatation suggesting evidence of high cardiac preload. Furthermore, there can be diastolic runoff from the patent ductus arteriosus toward the brachiocephalic arteries by color Doppler.

Endovascular treatment is the mainstay treatment for VGOM. Urgent endovascular treatment in the neonatal period is needed when medical management is not successful (74). The goal of treatment is to reduce shunt by 30% to reverse the PA pressure and reverse abdominal aorta flow, so that heart failure can be managed medically (31). High-output cardiac failure can result in renal or hepatic insufficiency. In older children and infants, treatment is targeted at reducing cognitive delays and cerebral atrophy. It is typically performed in a staged approach. Treatment can be done via a transarterial or transvenous fashion. However, transarterial embolization is the mainstay treatment approach at most centers, reserving transvenous embolization for cases in which transarterial embolization have been completely exhausted (73, 75, 76). Endovascular embolization results in a good clinical outcome and acceptable mortality and complications. Prior to endovascular intervention, the prognosis of this disease was 100% mortality without treatment (75).

In situations when hydrocephalus is not reversed post-embolization, it is treated with CSF diversion either via shunting or by endoscopic third ventriculostomy. In 2006 Lasjaunias et al. in a series of 233 patients treated with embolization reported the largest VGOM study (76). Mortality was 10.6%. Furthermore, 74% of those children who survived were neurologically intact, 15.6% were moderately developmentally delayed, and 10.4% were severely developmental delayed with a median follow-up of 4.4 years. Obliteration of 90–100% of the malformation was accomplished in 55% of patients using an arterial approach with glue embolization. In 2018 systemic review, Brinjikji et al. found that pediatric patients undergoing endovascular embolization of vein of Galen malformations demonstrated good long-term clinical outcomes in >60% of cases (77). In this study, the use of the Bicêtre neonatal evaluation score (BNES) resulted in improved rates of good neurologic outcome. One of the key observations from this review was that reports which utilized a predefined the BNES showed higher rates of good neurologic outcome than those that did not (76). The BNES is a 21-point score which assesses a combination of cardiac, respiratory, hepatic, renal, and neurologic functions (78).

## Section 4: Aneurysms

Children compromise 0.5–4.6% of all unruptured, asymptomatic aneurysms. Ruptured aneurysms are more rare with 0.6% of all aneurysmal SAH comprising pediatric patients <19 years of age (79–81). Children are more likely to present with aneurysms in the posterior circulation (25% children vs. 8% adults). They are also less likely to have anterior cerebral artery aneurysms (5% 10% in children vs. 34% adults) (79, 80, 82–84). Children are 2–4 times more likely to have giant (>2.5 cm) aneurysms than adults (79, 82, 85, 86).

Aneurysms appearance can be saccular (a focal outpouching of the vessel wall) or fusiform (caused by an injury to the vessel wall usually leading to a dilatation). Mycotic aneurysms are a different type of aneurysm usually caused by infection (rarely tumor) and often appear similar to dissecting aneurysms on angiogram. Younger children (<5 years old) often present with dissecting fusiform aneurysm whereas older children have saccular aneurysms (79, 82, 85). Most commonly they are saccular and account for 46–70% of aneurysms. Infectious aneurysms compose 5–15% of cases and are often are associated with severe cardiac disease and/or systemic infection. Genetic conditions associated with pediatric intracranial aneurysms include fibromuscular dysplasia, Marfan syndrome, polycystic kidney disease, Ehlers–Danlos syndrome, HHT, and Klippel–Trenaunay–Weber syndrome (79, 82, 83, 85–89). Table 3 demonstrates heritable disorders associated with an increased incidence of aneurysms in pediatric patients (90–120).

**Table 3:** Heritable disorders associated with intracranial aneurysms.

**Disorder**	**Inheritance**	**Special features**
α-Glucosidase deficiency	AR	Aneurysms often fusiform
α_1_-Antitrypsin deficiency	AR	Heterozygotes may be affected
Alkaptonuria	AR	
Anderson-Fabry disease	XLR	Aneurysms often fusiform; Carriers may be affected
Autosomal polycystic kidney disease	AD	
Ehlers-Danlos syndrome type IV	AD	
Familial idiopathic non-arteriosclerotic cerebral calcification syndrome	?	
Hereditary hemorrhagic telangiectasia	AD	
Marfan's syndrome	AD	
Neurofibromatosis type 1	AD	
Noonan's syndrome	AD	
Pseudoxanthoma elasticum	AD, AR	
Tuberous sclerosis	AD	Aneurysms often fusiform
Wermer's syndrome (multiple endocrine neoplasia type 1)	AD	
3M syndrome	?	

*AD, autosomal dominant; AR, autosomal recessive; XLR, X-linked recessive*.

Most aneurysms are asymptomatic and never found. Headache (80%) is the most common symptom. This is subsequently followed by loss of consciousness (25%), seizures (20%), focal neurological deficits (20%), and visual changes (10%) (79, 83, 85, 87). Non-invasive CT/MR angiography followed by DSA are usually performed to establish the diagnosis and best therapeutic approach.

**Figure 5:**
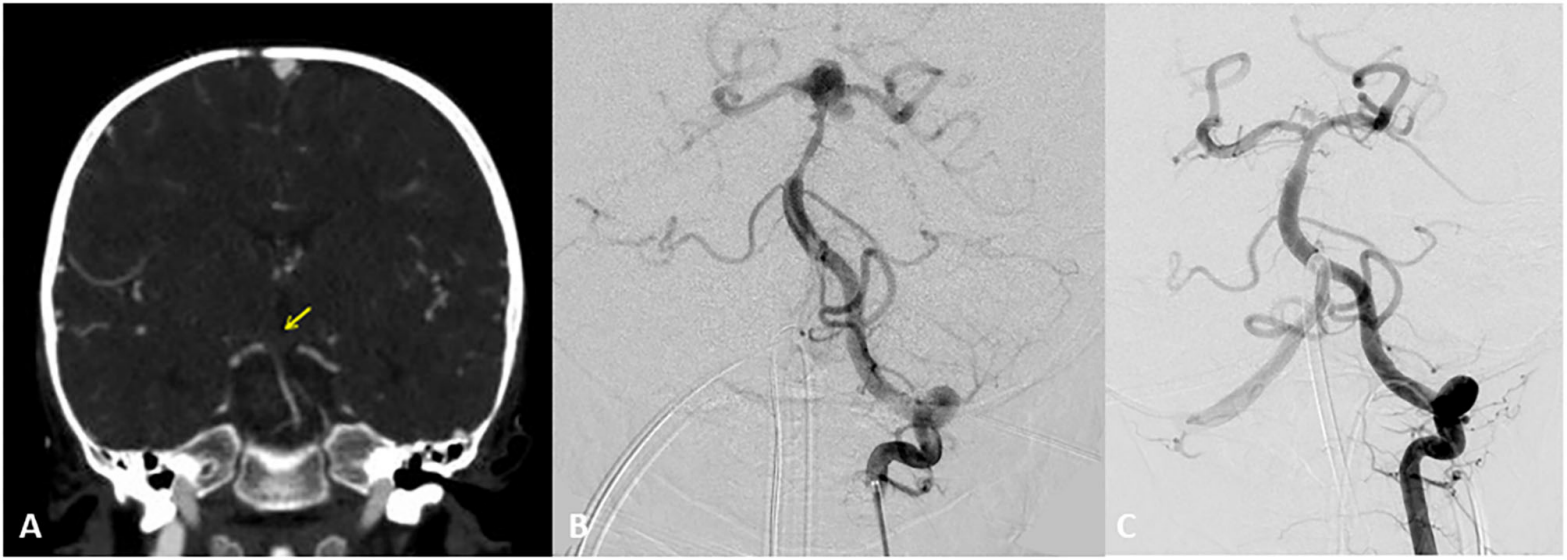
Two-year-old boy with hypoplastic left ventricle status post-cardiac repair presents with fevers, nausea, lethargy, and right sided weakness. Initial CT Angiogram coronal view revealed **(A)** revealed an acute clot on the top the basilar for which heparin and empiric antibiotics were started. Transthoracic ECHO revealed a large atrial mass and blood cultures revealed Gemella species. Follow up DSA AP view revealed at 2 weeks **(B)** depicted the development of a bilobular complex basilar tip aneurysm. After careful consideration a flow diverter stent was placed from left posterior cerebral artery to distal basilar artery. At the time of treatment a Pipeline flow diverter was utilized. Currently an alternative option for treatment for this type of wide necked aneurysm would be a WEB (woven endobridge) device. This is a sphere of woven wires appearing like a basket which fills the aneurysm and provides intrasaccular flow diversion. AP DSA view at 6 months **(C)** follow up revealed near complete resolution of the aneurysm. Patient underwent heart transplantation 2 months later. Yellow arrow indicates acute clot on the top the basilar.

The decision to treat an aneurysm is usually done with collaboration between open surgeons and neurointerventionalists. Endovascular therapy has transformed the treatment of pediatric aneurysms given its non-invasive nature when compared to open surgery. It is critical when possible to have cases reviewed by neurointerventionalists and open surgeons to come up with a balanced treatment strategy. In children lesions >3 mm in size usually warrant treatment. Mycotic aneurysms are initially treated with antibiotics and only intervened upon if they are persistent in follow up (Figure 5). Morbidity and mortality of treatment varies with age, aneurysm type, and presentation. Previous series found morbidity rates of 8–14% and mortality rates of 1–3% (87, 121). Follow-up of the aneurysm usually includes angiogram or non-invasive MRA at 6 months and then yearly for 5 years. Other institutions perform imaging every 3–5 years afterwards (122).

## Section 5: Cavernous Malformations

**Figure 6:**
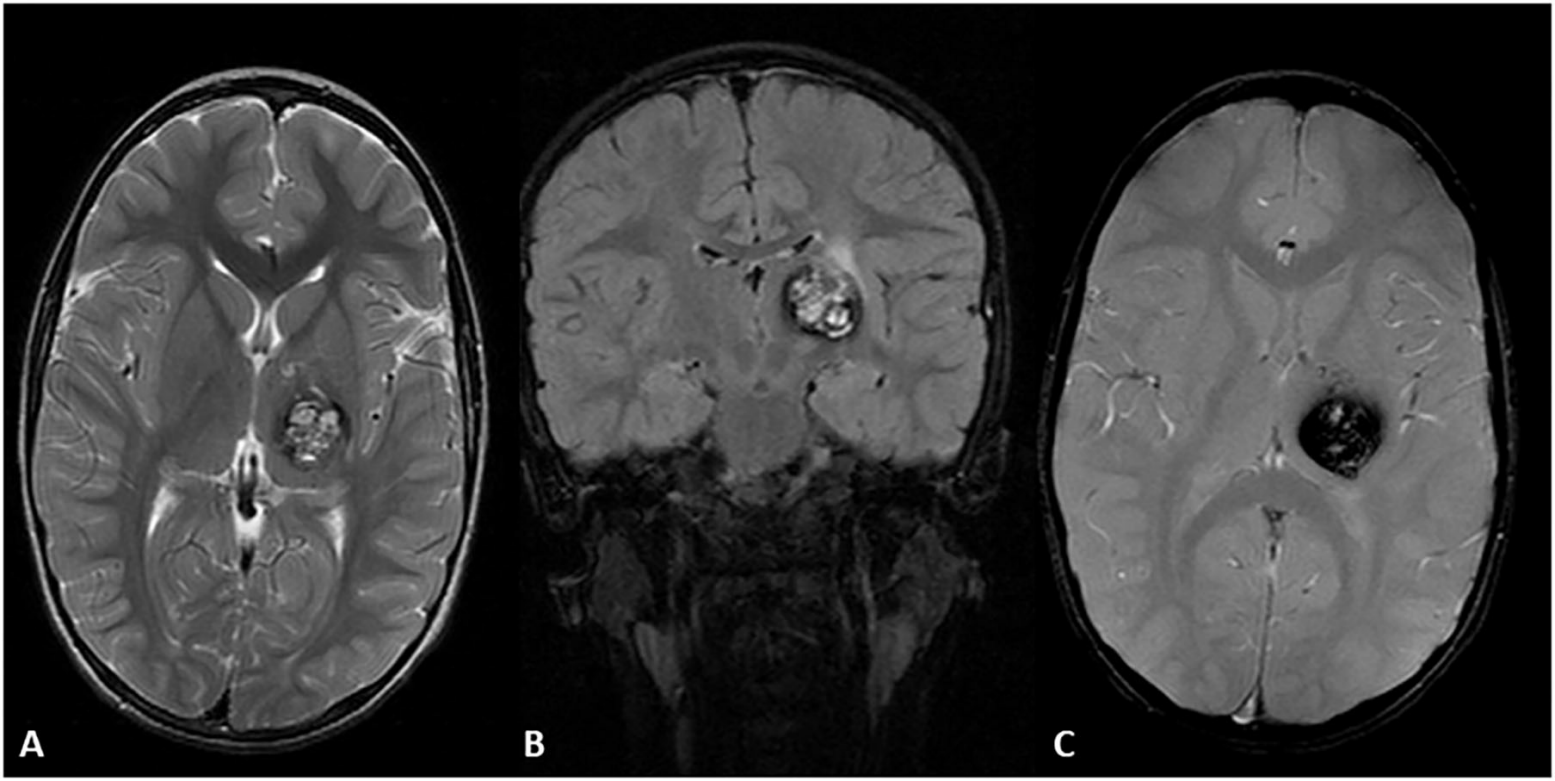
Four-year-old Hispanic girl presented with right sided tremor in the context of a new lesion compatible with cavernous malformation with subacute bleeding of the left thalamus. MRI axial T2 sequence **(A)** heterogenous hyperintense lesion with a hypointense rim due to subacute internal bleeding know as a “popcorn” appearance. MRI coronal FLAIR **(B,C)** demonstrates the same lesion with surrounding edema in the upper region of the lesion. MRI axial GRE T2 sequence demonstrates a prominent blooming artifact. T1 post-contrast showed no enhancement (not shown).

Cavernous malformations (CMs) are a type of cerebral vascular malformation (123). They are hamartomatous vascular lesions which macroscopically appear well-circumscribed, multilobulated with reddish purple color. Under the microscope they are seen as packed, enlarged, capillary-like vessels, without intervening parenchyma. Eighty percent of the lesions are supratentorial and the rest are infratentorial. The incidence of CMs in the general population varies from 0.1 to 0.9% with increased identification with advanced and widespread imaging modalities. CMs may form *de novo* and be sporadic, though there is higher association of multiple lesions with familial CMs. CMs have preponderance in the Hispanic population. Specifically, in pediatric population, they have been associated with history of radiosurgery for CNS neoplasms. Approximately one-fourth of patients in multiple series were children (124). Seizures are the most common presentation of patients with CMs. They occur in 40–50% of the patients and are likely due to their cortical location and possible irritation of surrounding parenchyma (124). Initial hemorrhage rate is 0.1–1.4% in sporadic lesions while 0.7–2.5% in familial lesions. The rebleeding risk has been reported as low as 0% in prospective studies and as high as 17% in retrospective studies. Infratentorial lesions tend to have a higher initial bleeding risk and rebleeding risk. CMs tends to be more aggressive in pediatric population and to have higher rates of growth and hemorrhage, atypical radiological features, and larger dimensions (125). Radiologically they have been classified into 4 types based on their appearance on MR imaging with colloquial description of “popcorn” appearance with a rim of signal loss secondary to hemosiderin [Figure 6; (126)]. CMs are angiographically occult vascular lesions. Conservative management with serial clinical and imaging follow up is the mainstay of treatment for CMs without gross hemorrhage or intractable seizures. It is also the default option for multiple asymptomatic CMs or surgically inaccessible or with high risk of morbidity. Otherwise surgical resection of CMs with or without neurophysiological monitoring has been well-reported in the literature. Surgical outcomes in most post-pediatric case series report ~0% mortality rate and a 4–5% rate of new permanent deficits (127, 128). Surgical resection of supratentorial lobar lesions yields high rates of symptomatic improvement with 98% resection rates and obliteration of seizures in 96% of patients (129). Radiographic follow-up is needed because CMs can recur. This can be seen in radiation-induced and familial cases (130, 131). Several centers order annual MRI studies for the initial 3–5 years after the resection with increasing intervals thereafter (129, 132). Radiosurgery of CMs remains controversial due to variable outcomes. Radiation therapy is used for surgically inaccessible targets demonstrating malignant natural history (133). Almost 25% of all cerebral cavernous malformations are linked with DVAs.

## Section 6: Developmental Venous Anomalies (DVAs)

The most common vascular malformations of the brain seen on routine imaging are developmental venous anomalies (DVAs). Fortunately, these lesions are often asymptomatic and incidentally found. DVAs are connected with parenchymal abnormalities and specific conditions like cerebral cavernous malformations. DVA angiographic architecture consists of a caput medusa and a medullary stem serving as a collecting vein. DVAs form the primary or only venous drainage for the area of the brain within their drainage territory. The caput medusa venous radicles are histologically defined by thin walled venous channels divided by normal brain parenchyma. The DVA collector is usually compromised of a thicker wall lacking a smooth muscle layer or elastica lamina (127, 128). Often, neuronal degeneration, gliosis, ischemic changes, and demyelination are demonstrated within the parenchyma (134–136). Based on previous autopsy studies the prevalence of DVAs is ~2.7% (137). However, recent imaging studies demonstrate the prevalence is higher at ~6.4% (138). Although the pathogenesis of DVAs is not completely understood, there is thought that to arise from intrauterine life. Some experts believe that DVAs are result from a cessation of normal venous development and a retention of primary medullary veins. Eventually this results in a recruitment of transparenchymal anastomotic drainage routes leading to development of a classic DVA draining into a superficial or normal deep vein. Some studies show that the angioarchitecture of DVAs may evolve during postnatal period and not during prenatal development (139, 140).

DVAs are typically asymptomatic. In a prospective cohort of 80 patients, McLaughlin et al. found a 0.68%/year risk of hemorrhage in DVAs (141). This risk is most likely related to associated cerebral cavernous malformations. Pereira et al. performed a study in adults and children and found following pathomechanisms responsible for symptoms: thrombosis and secondary infarction/hemorrhage (38%), associated arteriovenous shunting phenomena with or without hemorrhage (28%), mass effect from venous drainage causing hydrocephalus or cranial nerve injury (20%), and parenchymal injury/gliosis from increased venous pressure (6%) (142). DVAs are supratentorial in 70% or more of the time and infratentorial 14–19% of the time. The supratentorial areas which are more commonly affected are the frontal lobe (36–56%), parietal lobe (12–24%), temporal lobe (2–19%), and occipital lobe (4%). In infratentorial DVAs the most commonly affected region is the cerebellum (14–29%) with minority found in the brainstem (<5%) (143–145).

DVAs are usually found on CT and MRI imaging. They are usually not seen on non-contrast CT. However, in large DVAs a tubular focus is visible. This tubular focus can be confused with intracranial hemorrhage given its attenuation is similar to cerebral blood vessels. On contrast-enhanced CT and MRI, the transparenchymal vein is the most distinct feature. In smaller DVAs, an ill-defined contrast blush can be seen related to the venous radicles. On MR imaging, the collecting vein may show up as a flow void on T2 imaging. There is variability in the T2 signal related to the venous radicles. This is dependent on venous flow and acquisition angle. Venous radicles can demonstrate a hyperintense signal on fluid-attenuated inversion recovery (FLAIR) imaging. Susceptibility weighted imaging (SWI) is a sensitive method of detecting DVAs. This is because of the deoxygenated venous blood within the DVA, On SWI, the venous radicles and larger collecting vein are usually hypointense/dark (146, 147).

Horsch et al. (140) recently described DVAs in 14 neonates. In this series, DVAs (11/14) demonstrated hyperechoic parenchyma within the drainage territory. However, the reason for this elevated echogenicity remains to be elucidated. Most DVAs showed venous waveforms on doppler. A small proportion (4/14) demonstrated arterial-like waveforms. DVAs that are arterialized are typically rare. Horsch et al. (140) conjectured that this was secondary to arterialized DVAs being more common in earlier life and that they might lose arterialization later in life with vascular maturation. Early angiographic opacification is demonstrated an arterialized DVAs. The subtypes of arterialized DVAs have been identified type 1, caput medusae blush during arterial phase with no demonstrable arterial feeders or angiographically evident arteriovenous malformation (AVM) nidus; type 2, caput medusae blush during arterial phase with enlarged arterial feeders but no angiographically evident AVM nidus; type 3, DVAs draining an angiographically demonstrable AVM (148). The natural history of arterialized DVAs is remains to be elucidated. Treatment depends on clinical symptoms, parenchymal changes (edema/hemorrhage), and angioarchitecture (type). Radiotherapy, surgical, and endovascular approaches have been utilized. Typically, preservation of the DVA is attempted as it typically drains normal parenchymal (149).

## Author's Note

The aim of this review is to provide insight into pediatric hemorrhagic cerebrovascular disease. This article provides a thorough review of the diagnosis and management of pediatric cerebrovascular hemorrhagic pathology. A special emphasis is placed on neuroendovascular treatment options and several clinical vignettes are presented. This is topic is not frequently addressed and discussed among clinicians. Thus, this review will increase knowledge about this pathology and will empower practitioners to care for this unique patient population.

## Author Contributions

All authors listed have made a substantial, direct and intellectual contribution to the work, and approved it for publication.

## Conflict of Interest

The authors declare that the research was conducted in the absence of any commercial or financial relationships that could be construed as a potential conflict of interest.

## Abbreviations

ICH: intracerebral hemorrhage
SAH: subarachnoid hemorrhage
AVM: arteriovenous malformations
AV: arteriovenous
AVFs: arteriovenous fistulas
CM: cavernous malformations
DVA: developmental venous anomaly.
